# Hospital at Syracuse

**Published:** 1848-12

**Authors:** 


*Hospital at Syracuse.*—We perceive by a paper published at Syracuse, which has been forwarded to us, that the Common Council of that city have already taken steps to provide hospital accommodations in anticipation of the Cholera. This evinces a degree of prudence and humanity which other places would do well to imitate. Let proper sanitary regulations be at once adopted, and proper accommodations for the sick provided, and the visitation of Cholera, if not averted, we may reasonably hope, will be divested of much of its malignancy. We commend this subject to the attention of medical practitioners, whose duty it is to bring it to the notice of the proper anthorities.

